# Late-onset pattern macular dystrophy mimicking *ABCA4* and *PRPH2* disease is caused by a homozygous frameshift mutation in *ROM1*

**DOI:** 10.1101/mcs.a003624

**Published:** 2019-06

**Authors:** Chu Jian Ma, Winston Lee, Nicholas Stong, Jana Zernant, Stanley Chang, David Goldstein, Takayuki Nagasaki, Rando Allikmets

**Affiliations:** 1 Department of Ophthalmology, College of Physicians and Surgeons, Columbia University, New York, New York 10032, USA;; 2 Institute of Genomic Medicine, Columbia University, New York, New York 10032, USA;; 3 Department of Pathology and Cell Biology, Columbia University, New York, New York 10032, USA

**Keywords:** macular flecks, macular retinal pigment epithelial mottling

## Abstract

ROM1 (retinal outer segment membrane protein 1) is a 351-amino acid integral membrane protein on Chromosome 11q, with high structural similarity to PRPH2/RDS. Localized at the rims of photoreceptor outer segments (OSs), it is required for the maintenance of OS structure. Here, we describe a case with a phenotypic manifestation of a homozygous single-base pair deletion, c.712delC (p.Leu238Cysfs*78) in the *ROM1* gene, resulting in early termination at exon 2. The variant was detected by whole-exome sequencing (WES) in a 63-yr-old Caucasian woman with late-onset pattern macular dystrophy. Notably, although the phenotype resembles those caused by pathogenic variants in *ABCA4* or *RDS/PRPH2*, no pathogenic variants in these, or any other plausible candidate genes, were identified by WES. Clinical features include the presence of hyperautofluorescent flecks, relative sparing of the central macula, and preserved visual acuity. Reduced visual sensitivity was detected among flecked regions in the retina; however, full-field electroretinogram testing revealed no generalized cone dysfunction. The described first case of the complete loss of ROM1 protein function in the retina suggests its sufficiency for late-onset macular dystrophy. *ROM1* and *PRPH2* pattern macular dystrophies exhibit phenotype overlap, which may be attributable to their shared role in maintenance of the photoreceptor outer segment structure.

## CASE PRESENTATION

**Figure 1.**
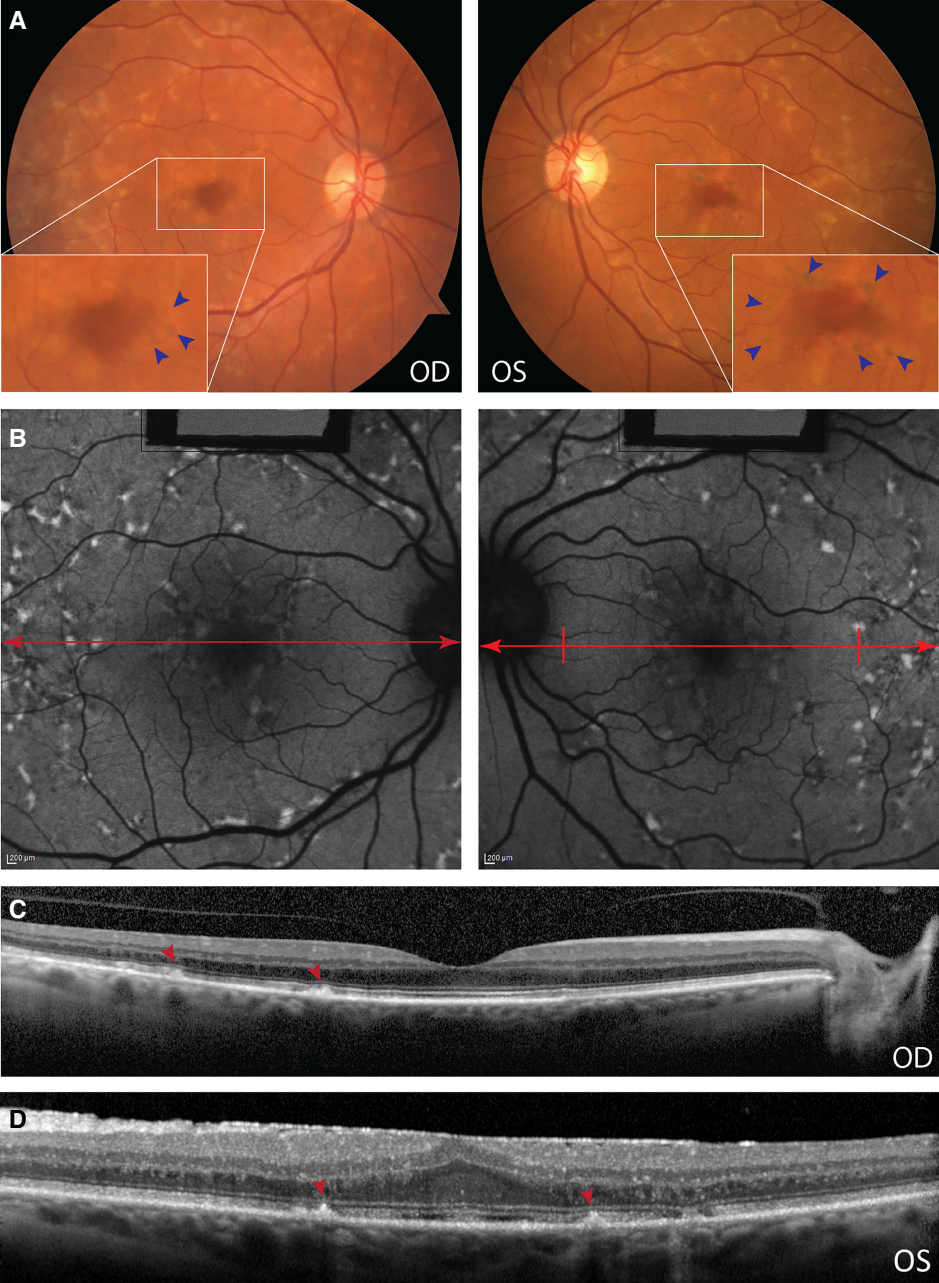
Retinal phenotype of a patient with pattern macular dystrophy due to homozygous frameshift mutations in *ROM1*. (*A*) Fundus photograph of left (*left*) and right (*right*) eyes showing diffuse yellow flecks in the macula extending to the mid-periphery. *Insets* with blue arrows point to pigment mottling in the parafoveal region. (*B*) Autofluorescence images showing the diffuse flecks as being autofluorescent with dark borders. Red arrows mark the level for the spectral-domain optical coherence tomography (SD-OCT) images shown *below.* (*C*) SD-OCT of the right eye showing FF flecks that originate from the RPE layer and that disrupt the photoreceptor integrity line and the outer limiting membrane. See red arrowheads. (*D*) SD-OCT of the left eye showing the loss of the foveal pit contour, indicating a prior epiretinal membrane.

A 63-yr-old Caucasian woman of Polish descent presented with a recent onset of visual symptoms. She was initially diagnosed with paramacular drusen associated with age-related macular degeneration (AMD). Best-corrected visual acuity (BCVA) was 20/25 OD and 20/40 OS and remained stable in the following 2 years. A history of smoking was noted, but no history of ocular trauma or inflammation was reported. A positive family history consisted of a brother diagnosed with advanced neovascular AMD. Slit-lamp examination of the anterior segment was within normal limits. Fundus examination revealed healthy optic nerves with no disk pallor and normal retinal vasculature appropriate for age without significant thinning or attenuation; there were diffuse yellow flecks in the peripheral macula extending out into the mid-periphery. Clustered, confluent patterns were observed in the temporal macula and arranged radially in the central macula. Pigment stippling/mottling around the fovea were also observed (Fig. 1A, inset, blue arrowheads).

Flecks were autofluorescent with dark borders. Spectral-domain optical coherence tomography (SD-OCT) revealed a loss of foveal pit contour, inner retinal thickening, and a hyper-reflective inner limiting membrane (ILM) in the left eye consistent with a developing epiretinal membrane. Flecks were visible as hyper-reflective deposits traversing photoreceptor layers emanating from the RPE (Fig. 1B). Ellipsoid zone (EZ) and external limiting membrane (ELM) layers are disrupted at the position of flecks (Fig. 1C, red arrowheads). Flecks of sufficient height impinged on the outer nuclear layer (ONL). Microperimetry (MP-1) testing (10-2 visual field pattern) showed reduced visual sensitivity and function over flecked areas (10–16 dB). Foveal fixation was stable (BCEA = 1.26 deg^2^) at 95.4% (Supplemental Fig. 1A). Full-field electroretinogram (ffERG) testing was conducted and analyzed in accordance with International Society for Clinical Electrophysiology of Vision (ISCEV) standards (McCulloch et al. 2015). Results from single flash cone and 30-Hz flicker stimuli showed normal waveform amplitudes and no implicit time delays indicating no generalized dysfunction of the cone system (Supplemental Fig. 1B,C). See Table 1 for a summary of the clinical findings.

**Table 1.** Clinical findings

Pattern dystrophy clinical features	Patient	Comments
Reduced visual acuity	+	20/25 OD and 20/40 OS
Retinal flecks	+	Diffuse yellow flecks in peripheral macula extending to mid-periphery
Retinal pigment epithelial mottling	+	Parafoveal pigment stippling/mottling
Epiretinal membrane	+	OS
ERG	–	
Microperimetry	+	Decreased visual sensitivities over flecked areas
Abnormal foveal pit on macular OCT	+	Loss of foveal pit contour
Choroidal neovascularization	–	

## TECHNICAL ANALYSIS AND METHODS

Genomic DNA was isolated from whole-blood lymphocytes. Sanger sequencing of *ABCA4* and *PRPH2* revealed no disease-causing mutations. WES and variant calling were performed at the Columbia Institute for Genomic Medicine (IGM). Exome sequence was captured using SeqCap EZ Exome v3. Raw sequence data was quality-filtered with CASAVA and aligned to Human Reference Genome build hg19; duplicates were removed with Dynamic Read Analysis for Genomics (Edico Genome), and variant calling was done according to best practices outlines in Genome Analysis Tool Kit (GATK v3.6) (McKenna et al. 2010) with variant selection using Variant Quality Score Recalibration using HapMap v3.3, dbSNP, and 1000 Genomes. Proband was sequenced to a mean coverage of 78× (see Supplemental Table 1 for metrics). A total of 307,946 variants were identified in this patient. To find possible disease-causing variants, the variants were filtered by allele frequency with the gnomAD database (MAF < 0.005), pathogenicity (known, or PolyPhen not benign), and consequence (missense, frameshift, splice donor/acceptor variants, and stop loss/gain), resulting in 221 variants. These variants were further filtered by mode of inheritance; as both of the patient's parents were not known to have any phenotype, we assumed that the disease-causing variants would be compound heterozygous or homozygous. Of the remaining eight distinct candidate variants from six genes, only the homozygous *ROM1* mutation was in a known retinal disease–associated gene (RetNet; https://sph.uth.edu/retnet/) (see Table 2). Of the other genes, titin (*TTN*) is a large abundant protein of striated muscle. *RYR1* encodes the ryanodine receptor 1, which is involved in calcium transport, important for skeletal muscle cells. *MUC4* is a mucin gene. *OR6C75* is an olfactory receptor. *DDX51* (DEAD-box helicase 51) is an ATP-binding RNA helicase involved in production of the 60S subunit of ribosomes. None of these genes has been associated with eye disease phenotypes, and there is no plausible mechanism on how they would be implicated in ocular phenotypes.

**Table 2.** Genomic findings

Gene	Chr	HGVS DNA	HGVS protein	Variant type and effect	dbSNP/dbVar ID	Genotype
*ROM1*	11	ENST00000278833: c.712delC	p.Leu238Cysfs*78	Frameshift deletion/premature stop codon	rs747855165	Homozygous

## VARIANT INTERPRETATION

The variant identified was a homozygous 1-bp deletion (11:62381846 AC/A, ENST00000278833, c.712delC, p.Leu238Cysfs*78; rs747855165) in exon 2 of the *ROM1* gene (see Table 2). This mutation had previously been reported in heterozygous carriers (Comander et al. 2017) and is especially prevalent in individuals of Ashkenazi Jewish descent at minor allele frequency MAF = 0.0029. It is practically absent (MAF < 0.0001) in other populations (http://gnomad.broadinstitute.org/variant/11-62381846-AC-A; accessed July 2018). The mutation results in a single-nucleotide deletion (at first base of codon 238, in exon 2 of the canonical transcript), changing leucine to cysteine CTG > TGT, with an early termination at amino acid position 78 (in exon 3). This new stop codon would occur in the last exon, and RNA therefore escapes nonsense mediated decay. The mutation harbored by our patient would rather result in the production of a truncated, likely nonfunctional, ROM1 protein with removal of one of the (terminal) cysteines involved in intramolecular disulfide bridges.

The pathophysiology of *ROM1*-associated pattern dystrophy (PD) is uncertain; however, the construction of a disease model would require a simultaneous consideration of *PRPH2* and *ROM1* as both function cross-dependently in OS biogenesis and maintenance (Zulliger et al. 2018). *Rom1*^−/−^ mice showed large discs and disorganized rod OS with slow degeneration of the rod photoreceptors by apoptosis (Clarke et al. 2000). It is also interesting to note that although the mice showed reduced ERGs at 12 mo, our patient's ffERGs are normal. In comparison, rod-dominated diseases due to variation in *PRPH2* are a consequence of haploinsufficiency (Kedzierski et al. 2001), as a result of either a null allele, as reflected by the rod degeneration phenotype of *Rds*^+/−^ mice, or a dominant negative (gain-of-function) allele. A case of the latter can be seen in the *Rds* p.Pro216Leu mice, in which levels of PRPH2 protein are depleted as a result of the degradation of the mutant protein (Kedzierski et al. 2001; Nour et al. 2004).

The disease mechanism of cone-dominated diseases due to *PRPH2* mutations is less certain, although most variants studied thus far exhibit three consistent features: (1) critical levels of PRPH2 are maintained (no haploinsufficiency); (2) both mutant and wild-type PRPH2 are properly trafficked to the outer segments; and (3) the pathogenicity of each mutation alters PRPH2/ROM1 complex formation. Because of the difference in PRPH2-ROM1 assembly and distribution between rods and cones, rods are more sensitive to the total level of PRPH2, whereas cones are more sensitive to proper formation of high-order complexes. In addition, the formation of PRPH2/ROM1 tetramers is mediated by an early-stage intermolecular disulfide bond between the PRPH2-Cys150/ROM1-Cys152 residues within the D2 loop only in cones (Zulliger et al. 2018). Removal of this cysteine residue, as with the p.Cys150Ser mutation, does not affect the formation of PRPH2 complexes but disrupts PRPH2/ROM1 complex assembly and OS localization of PRPH2 in cones (but not rods) (Chakraborty et al. 2009; Zulliger et al. 2018). Similarly to the Cys150Ser mutation, if our mutant prevents correct assembly of PRPH2-ROM1 complexes and higher-order PRPH2 complexes but not PRPH2 trafficking, a selective perturbation of cones is conceivable and would be consistent with the observed PD phenotype of the patient. The introduction of an additional cysteine residue (p.Leu238Cysfs*78) may also contribute to aberrant ROM1 binding and subsequent formation of excessively large PRPH2/ROM1 complexes as observed with the PD-associated p.Tyr141Cys variant of PRPH2 (Conley et al. 2017).

## SUMMARY

Retinal outer segment membrane protein 1 (*ROM1*, OMIM #180721) is a photoreceptor-specific integral membrane protein localized to the rim regions of rod and cone outer segments (OSs) (Bascom et al. 1992a,b; Moritz and Molday 1996). The function of ROM1 requires interaction with its structural homolog, peripherin 2 (*PRPH2*, or *RDS*, OMIM# 179605), with which it shares 55% nucleotide and 35% amino acid identity. Specifically, ROM1 and PRPH2 form complexes through both covalent and noncovalent interactions that are critical to the formation and maintenance of photoreceptor outer segments (Zulliger et al. 2018). More than 150 disease-causing mutations have been attributed to *PRPH2*, resulting in a spectrum of autosomal recessive and dominant retinal degenerations including central areolar choroidal dystrophy, pattern dystrophy, adult-onset vitelliform macular dystrophy, Leber congenital amaurosis (LCA), and retinitis pigmentosa (Farrar et al. 1991; Kajiwara et al. 1994; Wang et al. 2013). Although *Rom1*^−/−^ mice exhibit much milder phenotype than *Prph2* knockouts, and retinal disease attributable to pathogenic mutations in *ROM1* alone has yet to be described, ROM1 has been shown to play a significant role in OS biogenesis, especially in the maturation of the OS with PRPH2 (Sato et al. 2010; Zulliger et al. 2018).

Many prior studies have also suggested an interdependent role for PRPH2 and ROM1. For instance, the pattern dystrophy (PD) phenotype associated with the p.Tyr141Cys mutation of *PRPH2* (likely secondary to abnormal PRPH2-ROM1 complexes) can be converted to a haploinsufficiency RP phenotype in the complete absence of ROM1 (null alleles) (Conley et al. 2017). This highlights ROM1's different roles in the assembly of PRPH2 complexes between rods and cones. As such, the pathophysiology of *ROM1* loss of function mutants warrants continued inquiry especially in light of recent advances in gene discovery and clinical detection capabilities. This report identifies the first case of pattern macular dystrophy due to homozygous frameshift mutations in *ROM1* following the analysis of WES.

The retinal disease phenotype of the presented case exhibits features consistent with late-onset PD, including a butterfly-like central lesion associated with *PRPH2* and, more recently, recessive mutations in *CTNNA1* (Saksens et al. 2016). The presence of autofluorescent foci or flecks in the fundus is generally attributable to *ABCA4*-associated diseases (Sparrow et al. 2015). However, Stargardt or fundus flavimaculatus–like phenotypes of PD have been described in patients and mice harboring *PRPH2* mutations (Stuck et al. 2014), particularly in frameshift mutations (Boon et al. 2007) and in the diffuse, perimacular distribution of flecks as observed in our case (Boon et al. 2007; Duncker et al. 2015).

The phenotypic intersection between Stargardt disease and PD is also apparent from a histopathological study of a *PRPH2* p.Cys213Tyr PD donor eye, finding that RPE cells were distended with lipofuscin much in an analogous manner to donor eyes with defective ABCA4 (Zhang et al. 2002). In consistency with PD, both the late age of symptom onset (approximately seventh decade) and the preservation of the central macula are indicative of a relatively mild disease course in this patient, which is also shown by the slow rod degeneration seen in *Rom1* knockout mice (Clarke et al. 2000). However, severe vision loss due to chorioretinal atrophy and choroidal neovascularization (CNV) has been described and reported to be as high as 50% in individuals after 80 yr of age (Zhang et al. 2002; Boon et al. 2007). Although CNV was not observed at the time of examination, the patient reported a positive family history. Analyses of larger cohorts of confirmed *ROM1*-associated PD will be necessary to adequately characterize its phenotype; however, no other confirmed cases have been reported thus far. The paucity of documented cases could relate to the delayed onset of visual symptoms, which may preclude many affected individuals from presenting to the clinic, although it is also possible that many are misdiagnosed with AMD given the many overlapping features described above.

In summary, we identified the first case of *ROM1*-associated PD due to a homozygous frameshift mutation in *ROM1* without disease-causing mutations in *PRPH2* or any other gene. The clinical phenotype exhibits significant overlap with *PRPH2*-PD as well as other macular dystrophies, including AMD, which may explain the paucity of described cases in the literature.

## ADDITIONAL INFORMATION

### Data Deposition and Access

All sequence data and interpreted variants have been deposited in LOVD (Leiden Open Variation Database; www.lovd.nl), individual #00204287.

### Ethics Statement

Genetic screening was performed after informed consent and enrollment into the Columbia University Institution Review Board (IRB) study protocol #AAAI9906. All procedures were performed in accordance with the Declaration of Helsinki.

### Acknowledgments

We thank the patient for participation in this research.

### Author Contributions

All authors contributed to scientific discussion, variant interpretation, and manuscript review.

### Funding

This work was supported, in part, by grants from the National Eye Institute/National Institutes of Health EY028203 and EY019007 (Core Support for Vision Research) and unrestricted funds from Research to Prevent Blindness (New York, New York) to the Department of Ophthalmology, Columbia University.

### Competing Interest Statement

The authors have declared no competing interest.

### Referees

Robert Koenekoop

Roderick McInnes

Janey Wiggs

Kinga M. Bujakowskaa

## Supplementary Material

Supplemental Material
