# Supplementary material for: Late-onset pattern macular dystrophy mimicking ABCA4 and PRPH2 disease is caused by a homozygous frameshift mutation in ROM1
Source: Cold Spring Harb Mol Case Stud. 2019 Jun;5(3):a003624. doi: 10.1101/mcs.a003624 (PMC6549556; doi:10.1101/mcs.a003624)
Supplement: Supplemental Material [file supp_5_3_a003624__index.html]

Late-onset pattern macular dystrophy mimicking ABCA4 and PRPH2 disease is caused by a homozygous frameshift mutation in ROM1 — Supplemental Material 

# Late-onset pattern macular dystrophy mimicking *ABCA4* and *PRPH2* disease is caused by a homozygous frameshift mutation in *ROM1*

## Supplemental Material

- Supplemental\_Figure\_1.tif
- Supplemental\_Figure\_Legend.docx
- Supplemental\_Table.docx
