# Supplementary figures and images for: Late-onset pattern macular dystrophy mimicking ABCA4 and PRPH2 disease is caused by a homozygous frameshift mutation in ROM1
Source: Cold Spring Harb Mol Case Stud. 2019 Jun;5(3):a003624. doi: 10.1101/mcs.a003624 (PMC6549556; doi:10.1101/mcs.a003624)

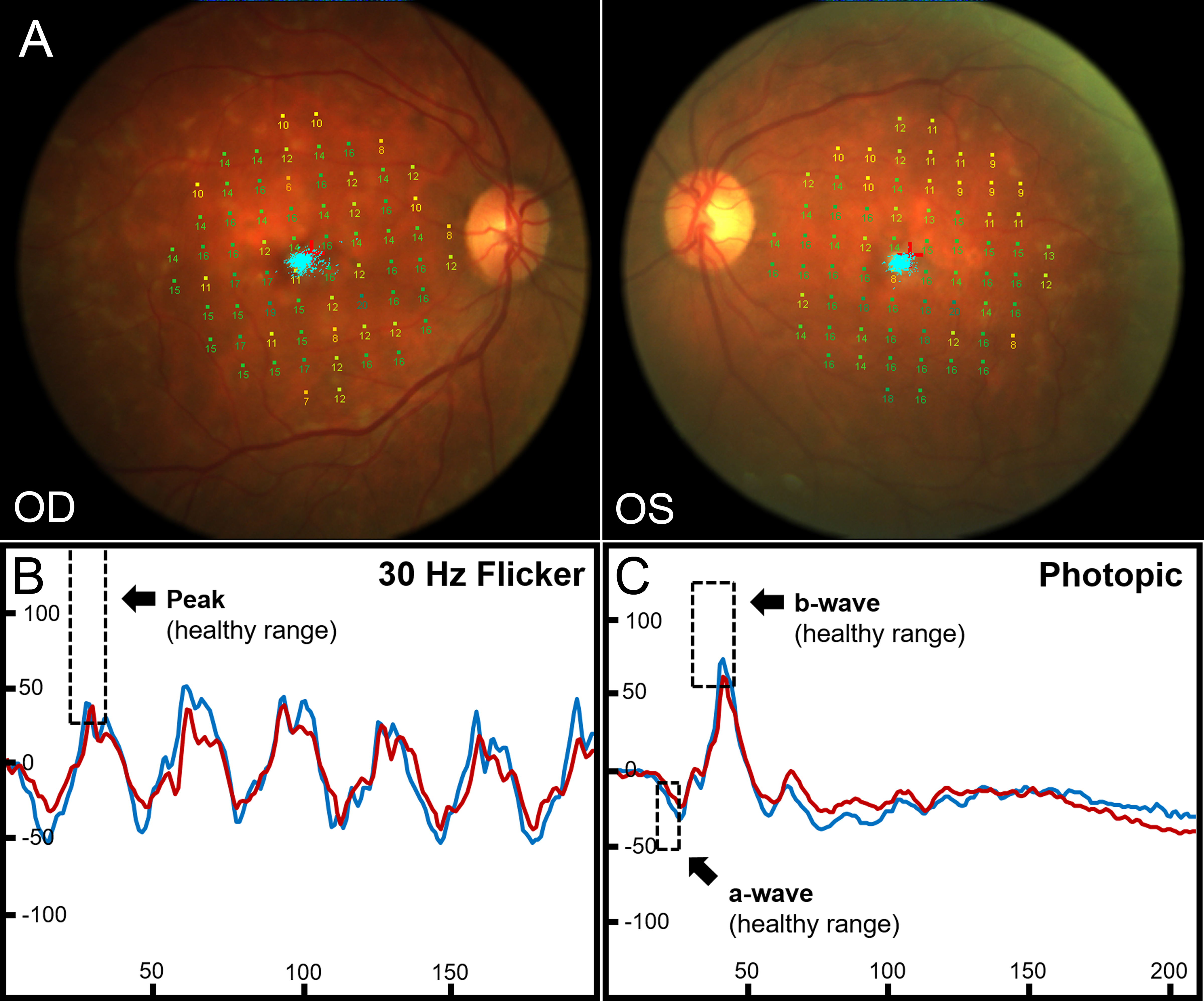

Supplement: Supplemental Material [file supp_mcs.a003624_Supplemental_Figure_1.tif]
