# Supplementary material for: Late-onset pattern macular dystrophy mimicking ABCA4 and PRPH2 disease is caused by a homozygous frameshift mutation in ROM1
Source: Cold Spring Harb Mol Case Stud. 2019 Jun;5(3):a003624. doi: 10.1101/mcs.a003624 (PMC6549556; doi:10.1101/mcs.a003624)
Supplement: Supplemental Material [file supp_mcs.a003624_Supplemental_Figure_Legend.docx]

**Supplemental Figure 1:** Macular and generalized retinal function in *ROM1* pattern macular dystrophy. (A) Microperimetry-1 (10-2) revealed relatively stable, central fixation and decreased visual sensitives around the fovea. (B) Photopic full-field electroretinogram testing revealed no decreased peak amplitude or latency to 30-Hz flicker and single flash stimuli relative to the normative range (dotted box).
