# Supplementary material for: Late-onset pattern macular dystrophy mimicking ABCA4 and PRPH2 disease is caused by a homozygous frameshift mutation in ROM1
Source: Cold Spring Harb Mol Case Stud. 2019 Jun;5(3):a003624. doi: 10.1101/mcs.a003624 (PMC6549556; doi:10.1101/mcs.a003624)
Supplement: Supplemental Material [file supp_mcs.a003624_Supplemental_Table.docx]

**Supplemental Table 1.** Exome sequencing metrics for patient described in this report

| Sample | Reads | Mapped | Duplicate | Map Rate | Dup. Rate | Target Depth |
| --- | --- | --- | --- | --- | --- | --- |
| Proband | 86048061 | 85921570 | 4388451 | 99.8% | 0.05% | 78.0X |
